# Supplementary material for: RNA modification landscape of the human mitochondrial tRNALys regulates protein synthesis
Source: Nat Commun. 2018 Sep 27;9:3966. doi: 10.1038/s41467-018-06471-z (PMC6160436; doi:10.1038/s41467-018-06471-z)
Supplement: Supplementary file 1 — Supplementary Information [file 41467_2018_6471_MOESM1_ESM.pdf]

Supplemental Information

RNA modification landscape of the human mitochondrial $tRNA^{Lys}$ regulates protein synthesis

Uwe Richter, Molly E. Evans, Wesley C. Clark, Paula Marttinen, Eric A. Shoubridge,
Anu Suomalainen, Anna Wredenberg, Anna Wedell, Tao Pan, and Brendan J. Battersby.

Supplemental Inventory
2 Figures

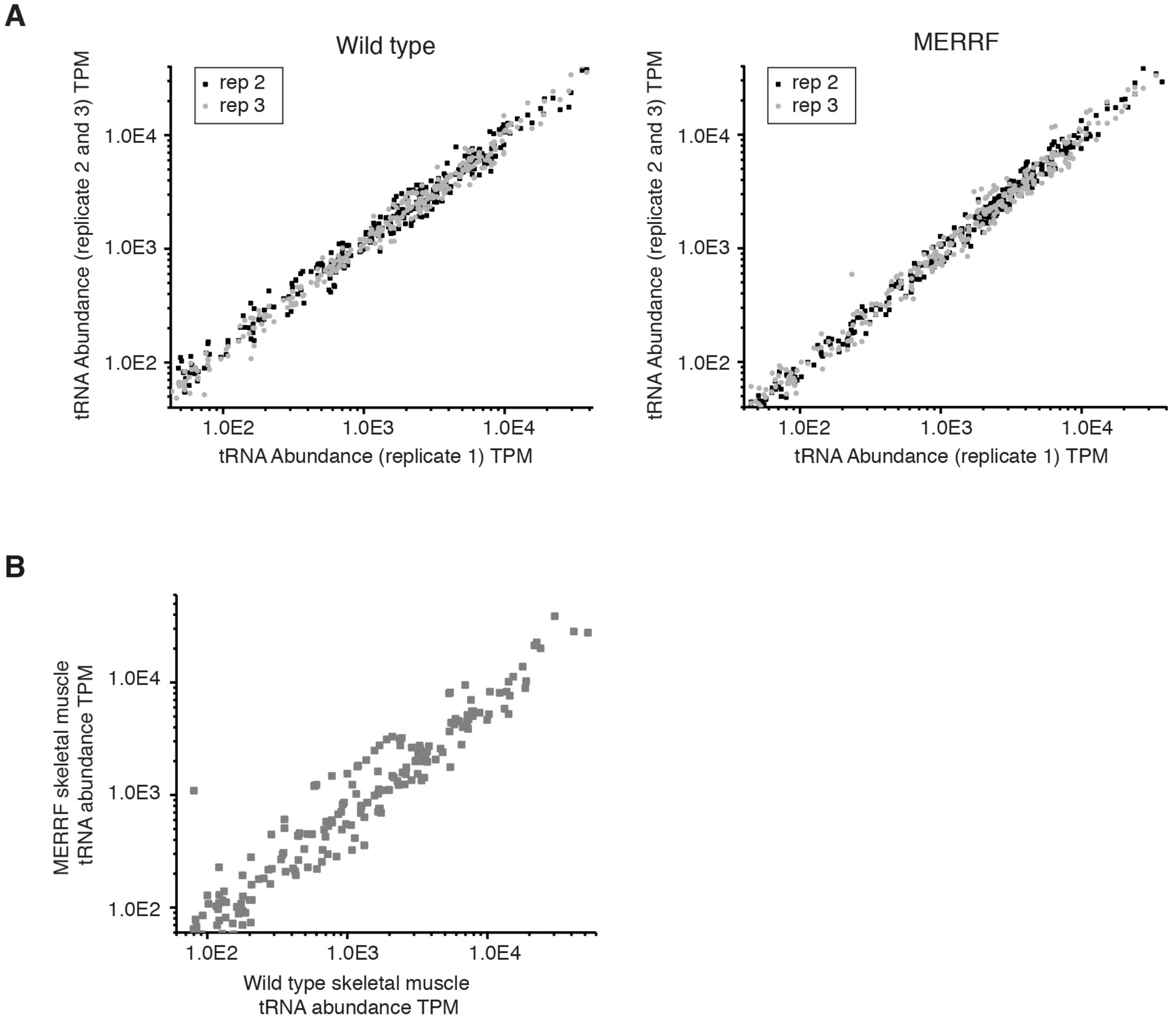

Supplementary Figure 1. Related to Figures 1 and 3.

(A) Number of uniquely aligned reads of mitochondrial tRNAs from human myoblasts homoplasmic for wild type or m.8344 A>G $tRNA^{Lys}$ MERRF mutation. Data is from three biological replicates (Pearson's coefficient >0.992 for all replicate pairs).

(B) Number of uniquely aligned reads of nuclear-encoded tRNAs from human skeletal muscle biopsies from a control and a patient diagnosed with the m.8344 A>G $tRNA^{Lys}$ MERRF mutation.

TPM = tRNA transcript per million

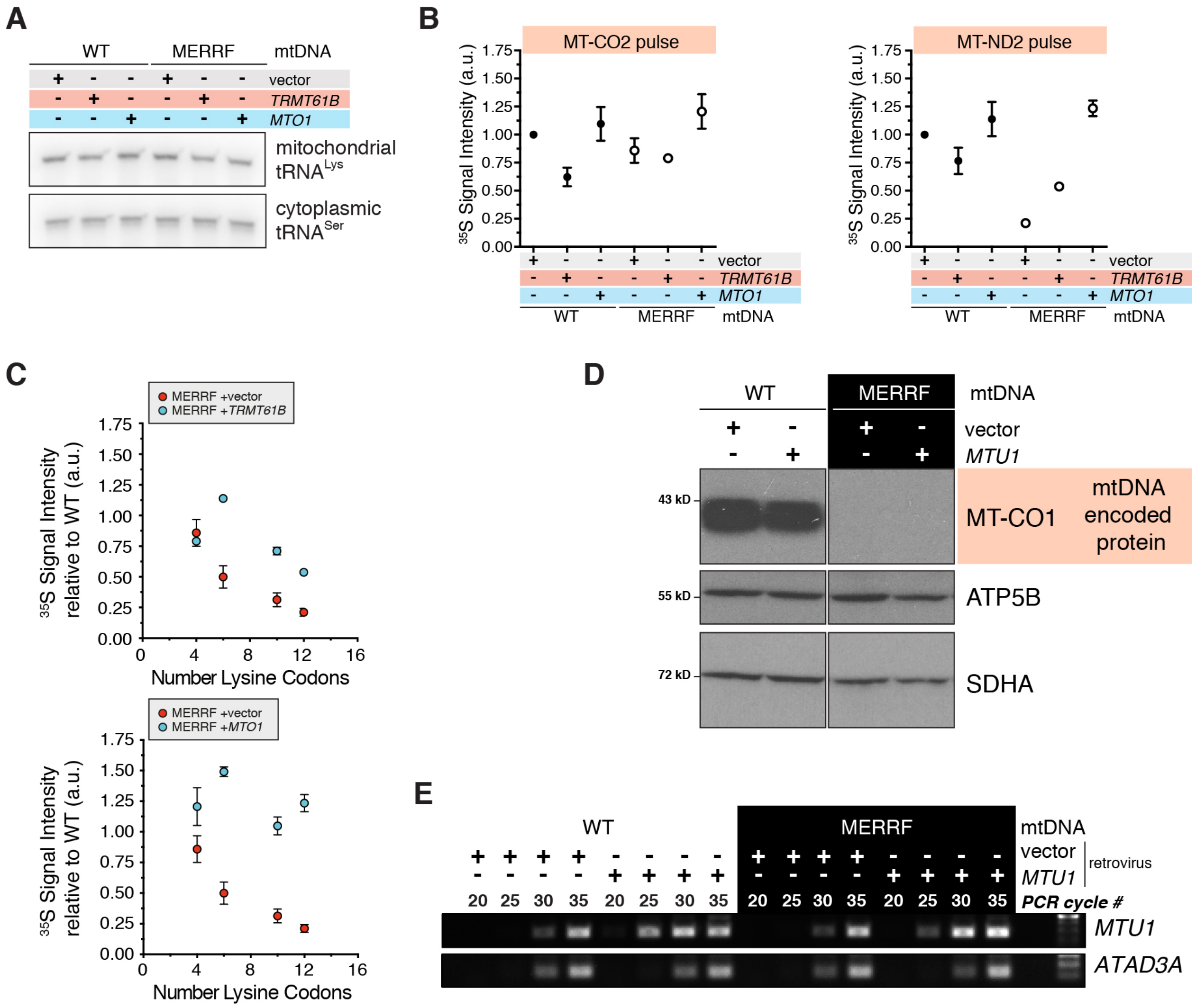

Supplementary Figure 2. Related to Figures 5.

(A) Northern blotting for tRNAs from human myoblasts homoplasmic for mitochondrial DNA wild type or with the m.8344 A>G $tRNA^{Lys}$ MERRF mutation following retroviral transduction with the indicated cDNAs.

(B) Quantification of ^{35}S incorporation into selected mitochondrial proteins during a 30 minute pulse (see Figure 5A). Data is mean +/- S.D. from three biological experiments.

(C) Quantified metabolic labeling of mitochondrial proteins during a 30 minute pulse of ^{35}S (mean +/- S.D., n=3) relative to the number of lysine codons in the respective polypeptides.

(D) Immunoblotting of whole cell lysates from human myoblasts homoplasmic for mitochondrial DNA wild type or with the m.8344 A>G $tRNA^{Lys}$ MERRF mutation following retroviral transduction with the indicated cDNAs.

(E) A representative semi-quantitative RT-PCR of total RNA isolated from myoblasts in (D) to assess *MTU1* cDNA overexpression.
